# Unravelling the combined effects of drought and nitrogen addition on carbon assimilation and reserves in Korean pine saplings

**DOI:** 10.3389/fpls.2025.1574468

**Published:** 2025-04-14

**Authors:** Jinyuan Tian, Hongxia Zhang, Anzhi Wang, Jiabing Wu, Sabine Rosner, Kai Zhu, Rongrong Cai, Fenghui Yuan

**Affiliations:** ^1^ CAS Key Laboratory of Forest Ecology and Silviculture, Institute of Applied Ecology, Chinese Academy of Sciences, Shenyang, China; ^2^ College of Resources and Environment, University of Chinese Academy of Sciences, Beijing, China; ^3^ Key Laboratory of Ecological Safety and Sustainable Development in Arid Lands/Shapotou Desert Research and Experiment Station, Northwest Institute of Eco-Environment and Resources, Chinese Academy of Sciences, Lanzhou, China; ^4^ Institute of Botany, Department of Ecosystem Management, Climate and Biodiversity, BOKU University, Vienna, Austria; ^5^ College of Resources and Environment, Shanxi Agricultural University, Taigu, China; ^6^ Department of Soil, Water, and Climate, University of Minnesota, Saint Paul, MN, United States

**Keywords:** drought, nitrogen deposition, combined abiotic stress effects, conifers, nonstructural carbohydrates

## Abstract

Climate change profoundly impacts the physiological processes and adaptation strategies of plants. However, the physiological mechanisms of coniferous species responding and adapting to combined drought and nitrogen (N) addition remain unclear. Here, based on 2-year multi-level N addition and drought experiments, we investigated the responses of carbon assimilation (net photosynthetic rate *A*_n_, stomatal conductance *g*_s_ and intrinsic water use efficiency WUE_i_) and carbon reserves (non-structural carbohydrates, NSC) of 7-year-old Korean pine (*Pinus koraiensis*) saplings. Our results showed that: (1) Drought decreased *A*_n_ and *g*_s_, while N addition increased *A*_n_ and decreased *g*_s_. N addition decreased *A*_n_ and WUE_i_ but increased *g*_s_ in plants under drought conditions, indicating that N addition under drought stress will maintain gas exchange by increasing stomatal opening, but failed to mitigate the reduction of *A*_n_. (2) Both drought (moderate and severe) and N addition reduced leaf NSC concentrations. Under moderate drought stress, however, N addition led to an increase in leaf NSC concentrations. (3) The interconversion between leaf starch and soluble sugars slowed the decrease in carbon assimilation caused by drought. *P. koraiensis* saplings adopted a conservative strategy of increasing leaf mass per area (LMA) to adapt to reduced water use efficiency. The study highlights the coordinated relationship between carbon assimilation and carbon reserves of Korean pine saplings under combined drought and N addition, which improves our understanding of the diverse carbon dynamics of different species under climate change.

## 1 Introduction

Forest ecosystems are the largest terrestrial carbon sink on Earth (Pan et al., 2011), and they are also critical habitats for biodiversity (Hisano et al., 2018). However, forest ecosystems are facing big challenges posed by climate change, such as prolonged severe droughts with warming (Anderegg et al., 2019; Sánchez-Pinillos et al., 2022) and increasing nitrogen (N) deposition due to large anthropogenic emissions of reactive N (Davidson, 2009; Roth et al., 2022), potentially limiting forest development and persistence, and thus on forest ecosystem services.

The ability of trees to assimilate carbon through photosynthesis is mostly highly sensitive to soil water-stress condition. During soil water stress, trees typically reduce water loss through transpiration by regulating leaf stomatal conductance (*g*_s_). This, in turn, leads to a decrease in the net photosynthetic rate (*A*_n_) as the rate of CO_2_ diffusion decreases, ultimately resulting in reduced CO_2_ assimilation (Rowland et al., 2015; Zhang et al., 2023). Excessive environmental stress can lead to a depression in photosynthesis, resulting in a diminished production of non-structural carbohydrates (NSC) that are crucial for respiration, growth, and defense. When the availability of NSC falls below the threshold required to sustain these vital functions, trees may succumb to carbon starvation (McDowell and Sevanto, 2010). Water stress can also alter the relationship between carbon assimilation and carbon storage in trees, reflecting the coordination of carbon acquisition and expenditure (Tsuji et al., 2022; Zhang et al., 2021b), the tree’s metabolic growth and survival ability (Li et al., 2022; Peltier et al., 2023), and its buffering capacity to withstand external stressors (Piper and Paula, 2020).

Unlike the negative impacts of soil water stress, N deposition often has positive impacts on trees, mainly by altering the N nutrient status of the soil. Recent meta-analyses have indicated that, in terms of carbon assimilation, N addition significantly increased leaf photosynthetic rate and intrinsic water use efficiency (WUE_i_), resulting in gross photosynthetic carbon gain (Liang et al., 2020; Zhang et al., 2018). In terms of carbon reserves, N addition also affects NSC concentrations and the allocation of photosynthetic assimilates in tree tissues, showing that carbon allocation for growth and respiration is stronger than that for NSC storage, and carbon allocation to aboveground parts is more than to belowground parts (Li et al., 2020). However, under N-saturated conditions, additional N input may attenuate this positive effect. For instance, studies have indicated with the accumulation of continuous N addition, there is a non-linear increase in carbon assimilation of conifer species such as Chinese fir *Cunninghamia lanceolata* (Li et al., 2022), and angiosperm species such as *Fraxinus mandshurica* (Wang et al., 2018). Furthermore, the NSC allocation of angiosperm and coniferous tree species to N addition differ, where *Pinus massoniana* allocates more NSC to roots, while *Castanopsis chinensis* to branches (Huang et al., 2021).

Although the responses of tree carbon assimilation and carbon storage to independent water stress or N addition have been extensively addressed, there is no clear consensus on their combined effects. Some studies have shown that N addition can offset the negative impacts of drought, for both coniferous and broadleaf tree species. For example, N addition (46 kg N ha^-1^ yr^-1^) improved carbon exchange and reserves traits (such as *A*_n_, *g*_s_, and leaf NSC) in two broadleaf species (*Quercus mongolica* and *Fraxinus mandshurica*), mitigating the negative impacts of drought (Zhang et al., 2021a). Under drought conditions, N addition (50 kg N ha^-1^ yr^-1^) positively affected *A*_n_ and WUE_i_ in the coniferous species of *Abies fabri* (Yang et al., 2012). On the other hand, some studies have suggested that the vulnerability of trees to drought will increase under N addition. For example, N addition (50 kg N ha^-1^ yr^-1^) increased aboveground biomass investment in European beech seedlings (*Fagus sylvatica*), but enhanced drought sensitivity, as evidenced by higher tissue mortality (Dziedek et al., 2017). The comprehensive effects depend on the combined levels of drought and N addition. For instance, N addition (80 kg N ha^-1^ yr^-1^) increased xylem soluble sugar and starch of two broadleaf species (*Ormosia pinnata* and *Schima superba*) under moderate drought (60% of soil field capacity), but decreased the xylem soluble sugar under severe drought (40% of soil field capacity) (Li et al., 2021). Recent study showed that the negative effects of droughts on the mortality of Norway spruce (*Picea abies*) would be enhanced with a critical threshold for N deposition of 10.9 kg N ha^-1^ yr^-1^ (Tresch et al., 2023).

Tree species with different drought tolerances also experience different combined impacts of water stress and N addition. On the one hand, the ability of plants to maintain carbon balance also depends significantly on the differences in stomatal regulation to drought stress (Henry et al., 2019), whereas gymnosperms and angiosperms have different response mechanisms. Leaf abscisic acid (ABA) levels play a major role in stomatal closure in angiosperms’ response to drought stress, while effective stomatal closure in gymnosperms is driven by a combination of leaf ABA levels and water potential (Brodribb and McAdam, 2013; McAdam et al., 2014). However, most studies have been focused on the responses of angiosperm tree species to the combined effects of different water stresses and N addition levels, with little attention on conifer tree species (Dulamsuren and Hauck, 2021).

In this study, in order to examine the physiological mechanisms underlying the responses of the temperate conifer tree species *Pinus koraiensis* to soil water stress and N deposition, we designed a field manipulation experiment with independent and combined drought and N addition. Leaf gas exchange (*A*_n_, *g*_s_ and WUE_i_), carbon reserves (NSC concentrations and its composition) and physicochemical characteristics of stem and leaf were measured. The study aimed to answer three questions: (1) What are the differences between the independent and combined treatments of soil water stress and N addition on gas exchange and carbon reserves of *P. koraiensis*? (2) Will the same effect exist at different combined levels of water stress and N addition? (3) Will the reduction in carbon assimilation be regulated by carbon reserves underlying interactive stresses?

## 2 Materials and methods

### 2.1 Description of study site

The experiment was conducted in Changbai Mountain Forest Ecosystem Research Station of Chinese Academy of Sciences (128° 28’E, 42° 24’N), which has a temperate continental mountain climate affected by monsoon. Average annual temperature is 3.6°C, and the average annual precipitation is 745 mm. The growing season is from late May to early October. Broadleaved Korean pine forest is a typical forest in the area, and *Pinus Koraiensis*, *Fraxinus mandshurica*, *Acer mono*, *Quercus mongolica* and *Tilia amurensis* are its dominant species. The soil in the forest is mainly dark brown soil with a soil field capacity of 85% (0-10 cm depth) (Peng et al., 2019). Average annual atmospheric N deposition in the Changbai Mountain region is 23 kg N ha^-1^ yr^-1^, and the rate of N deposition is expected to double by 2050 (Dong et al., 2022).

### 2.2 Experimental design and treatments

Seven-year-old *P. koraiensis* saplings of similar height (approximately 90 cm) were selected from field in 2017, and then transplanted in pots (diameter 41 cm × height 28 cm) in April of the same year for a two-year (from May to October in 2017 and 2018) multi-level N addition and drought experiment. The soils inside the pots were natural topsoil collected from the broadleaved Korean pine forest. There were three drought treatments: well-watered (maintained at soil field capacity), moderate drought (40-50% of soil field capacity) and severe drought (20-30% of soil field capacity). Under each drought treatment four N addition (urea solutions with different N addition concentrations were sprayed once a month) levels were conducted to simulate the atmospheric N deposition ranging from no N (N0, 0 kg N ha^-1^ yr^-1^), low N (N1, 23 kg N ha^-1^ yr^-1^), medium N (N2, 46 kg N ha^-1^ yr^-1^) and high N (N3, 69 kg N ha^-1^ yr^-1^) additions, respectively. A total of 36 tree saplings, representing 12 treatments in total, were used in the experiment. For each treatment, there were three replicates (n = 3) per species, with saplings randomly placed under a transparent rain shelter. Each sapling was measured three times with measurements averaged within each replicate to avoid pseudoreplication. Soil moisture in the pots was monitored at 30-min intervals automatedly using soil moisture sensors (Stevens Hydraprobe, Stevens Water Monitoring Systems, Inc., Portland, OR, USA). The changes in soil water content during the experiment are shown in **Supplementary Figure S1**. In order to distinguish the independent and combined effects of drought and N addition on plants, we defined the single N addition treatment as four N addition gradients under well-watered conditions, and the single drought treatment as three drought gradients without N addition.

### 2.3 Gas exchange

In late July 2018, the instantaneous values of leaf gas exchange parameters such as *A*_n_ and *g*_s_ of each pot were measured from 09:00 to 11:00 using a portable photosynthetic system (LI-6400XT, LI-COR Inc., Lincoln, NE, USA). Each sapling was measured three times with three different randomly selected needles. The average of these three measurements represents the measurement data for this plant. During the measurements, chamber CO_2_ concentration was set at environmental 400 ppm, photosynthetically active radiation was maintained at 1200 μmol m^-2^ s^-1^, the flow rate was 500 μmol s^-1^, and the leaf chamber temperature was maintained at 25 ± 1.5°C. Intrinsic water use efficiency (WUE_i_) was calculated using *A*_n_ and *g*_s_ (WUE_i_ = *A*_n_/*g*_s_).

### 2.4 Physicochemical characteristics and non-structural carbohydrates

After gas exchange measurements, measured needles were collected and scanned for leaf area using ImageJ software, and then dried to constant weight at 65°C to obtain leaf dry mass. Leaf mass per area (LMA, g m^-2^) was calculated as leaf dry mass/leaf area. Total C and total N (%) were determined using an elemental analyzer (Elementar Vario MACRO Cube, Hesse, Germany). The contents of soluble sugar (%) and starch (%) were determined by anthrone method described in Zhang et al., (2021a). NSC (%) is the sum of soluble sugar and starch concentrations.

### 2.5 Statistical analysis

The least significant difference method (LSD) of one-way ANOVA was used to compare the mean values to test the differences in gas exchange, carbohydrate characteristics and physicochemical characteristics under the same drought treatment or N addition level. Two-way ANOVA was used to analyze the effects of different levels of independent N addition, drought stress and their interaction on functional traits. Linear regression was used to analyze the relationships between parameters. Data are provided as mean ± SE. SPSS 21 was used for statistical analysis.

## 3 Results

### 3.1 Responses of gas exchange

**Figure 1:**
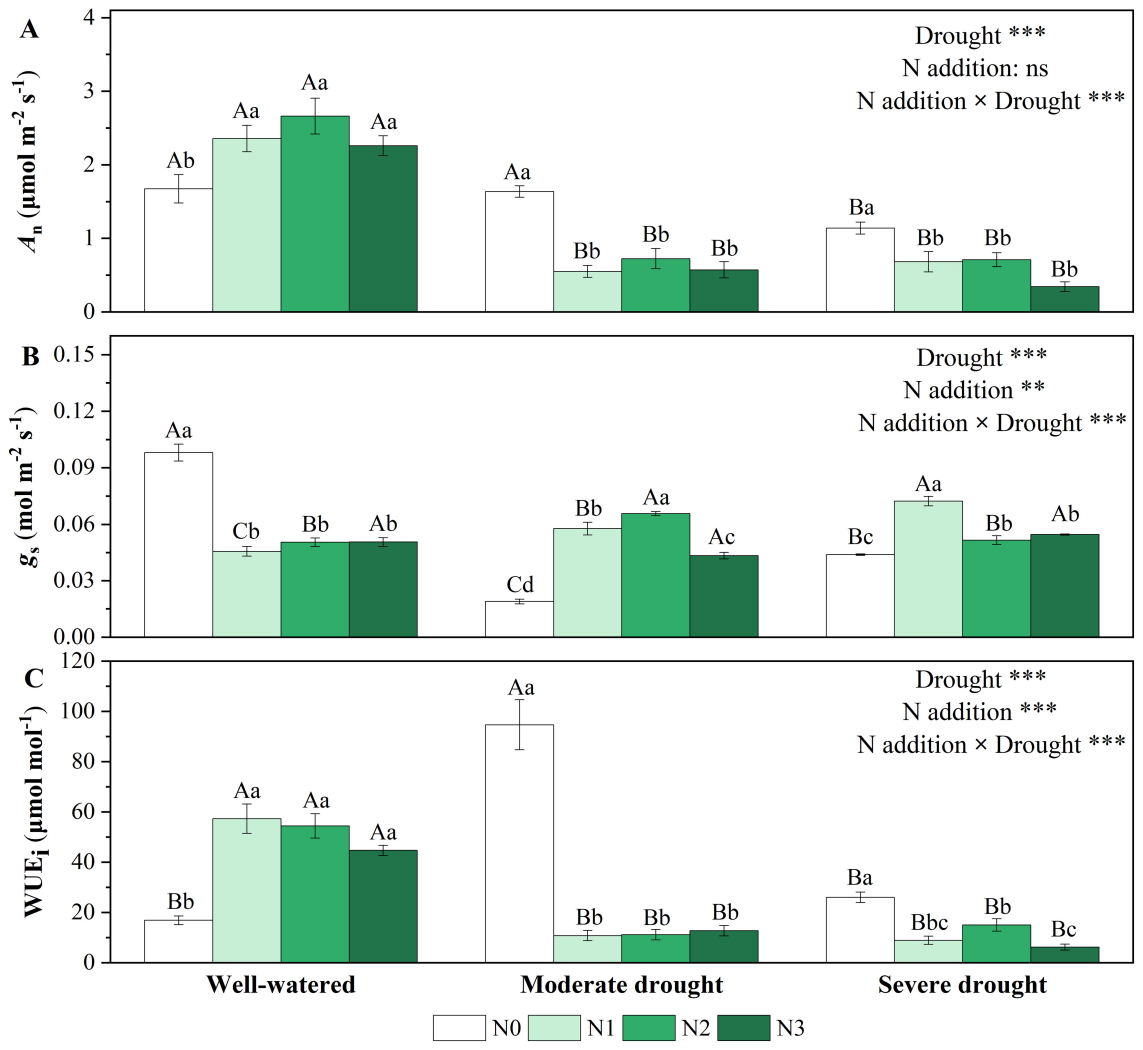
Responses of net photosynthetic rate [**(A)**, *A*_n_, μmol m^-2^ s^-1^], stomatal conductance [**(B)**, *g*_s_, mol m^-2^ s^-1^], and intrinsic water use efficiency [**(C)**, WUE_i_] of *P. koraiensis* to different treatments of soil water stress and N addition. Different uppercase letters indicated significant differences among different drought treatments at the same N addition level, and different lowercase letters indicated significant differences among different N addition levels in the same drought treatment (*P* < 0.05). n = 3. ***p < 0.001, **p < 0.01, *p < 0.05. N0-N3 represent N addition levels at 0, 23, 46, and 69 kg N ha^-1^ yr^-1^ respectively.

Drought and N addition treatments had different effects on gas exchange (**Figure 1**; **Supplementary Table S1**). Single drought treatments significantly reduced *A*_n_ and *g*_s_, reaching minimum values (1.139 ± 0.077 μmol m^-2^ s^-1^, 0.019 ± 0.001 mol m^-2^ s^-1^) at severe drought and moderate drought respectively (**Figures 1A, B**). WUE_i_ showed a trend of increasing and then decreasing with the increase of drought stress, reaching maximum values (94.677 ± 9.935 μmol mol^-1^) at moderate drought (**Figure 1C**). Single N addition treatments significantly increased *A*_n_ and WUE_i_ but decreased *g*_s_. Under drought stress, N addition treatments decreased *A*_n_ and WUE_i_, but increased (the already quite low) *g*_s_ (**Figure 1**). Medium N (N2) and low N (N1) addition had the strongest promoting effects on *g*_s_ under moderate and severe drought, respectively (**Figure 1B**).

### 3.2 Responses of carbon reserves

**Figure 2:**
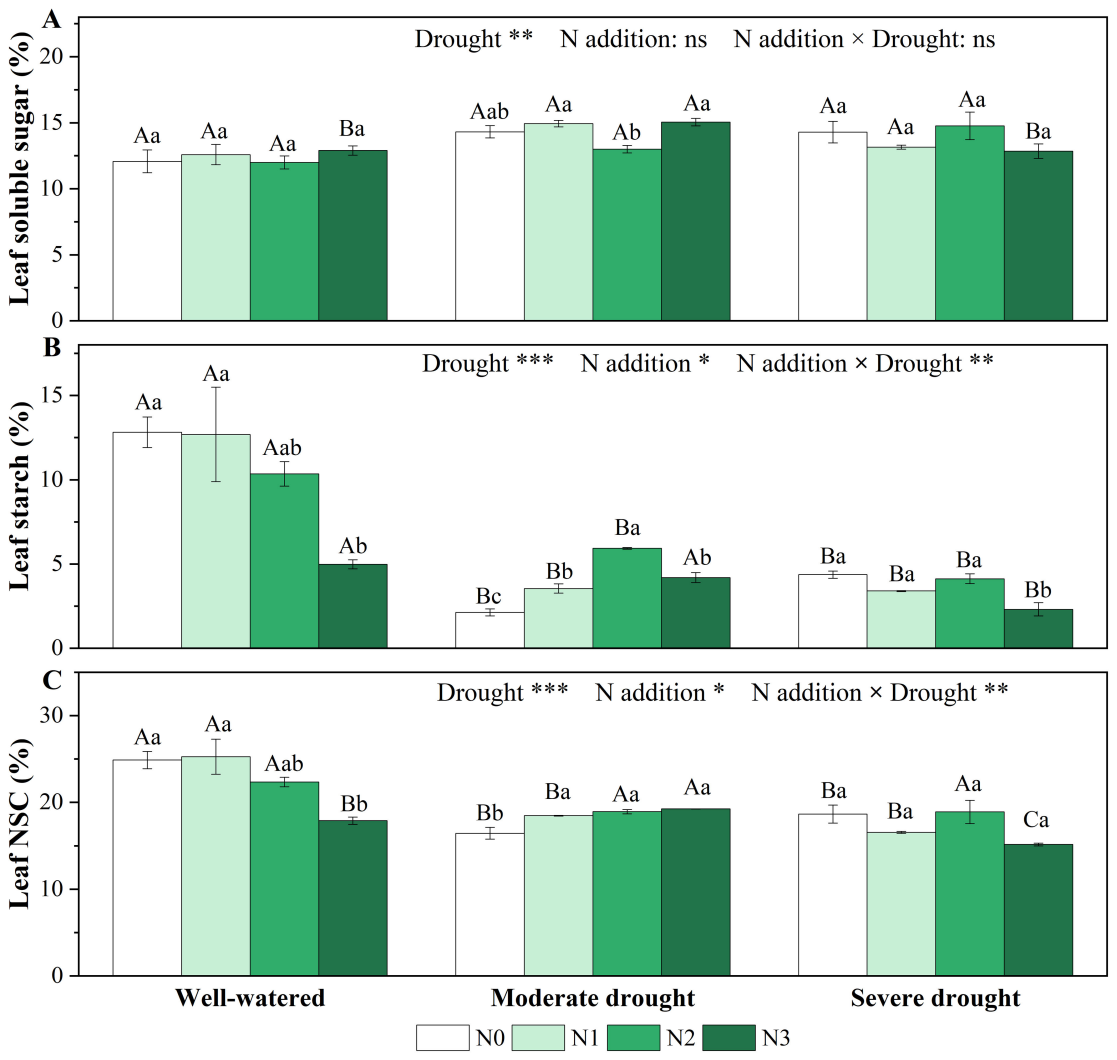
Responses of soluble sugar [**(A)**, %], starch [**(B)**, %], and non-structural carbohydrates [**(C)**, NSC, %] in the needles of *P. koraiensis* to different treatments of soil water stress and N addition. Different uppercase letters indicated significant differences among different drought treatments at the same N addition level, and different lowercase letters indicated significant differences among different N addition levels in the same drought treatment (*P* < 0.05). n = 3. ***p < 0.001, **p < 0.01, *p < 0.05. N0-N3 represent N addition levels at 0, 23, 46, and 69 kg N ha^-1^ yr^-1^ respectively.

Single drought and N addition treatments did not significantly affect the leaf soluble sugar concentrations, but significantly reduced leaf starch and NSC concentrations (**Figure 2**). Under moderate drought, N addition significantly increased leaf starch and NSC concentrations, with the effect on starch concentrations reaching its maximum at medium N addition (N2). However, this positive effect diminished under severe drought (**Figures 2B, C**). Overall, the interaction effects of drought and N addition on starch and NSC concentrations were significant in needles (**Figure 2**; **Supplementary Table S1**).

Unlike the needles, there were significant differences in the effects of all drought and N addition treatments on carbon reserves in stems (**Supplementary Figure S2**, **Supplementary Table S1**). Single drought treatments had no effect on soluble sugar but significantly increased stem starch and NSC concentrations. Single N addition treatments significantly increased starch concentrations only at high N addition (N3) (**Supplementary Figure S2B**). Under moderate drought, N addition had opposite effects on soluble sugar (increase) and starch concentrations (decrease), resulting in non-significant changes in NSC concentrations. Under severe drought, N addition had no statistically significant effect on soluble sugar but significantly decreased starch and NSC concentrations (**Supplementary Figure S2**).

### 3.3 Responses of physicochemical traits

**Figure 3:**
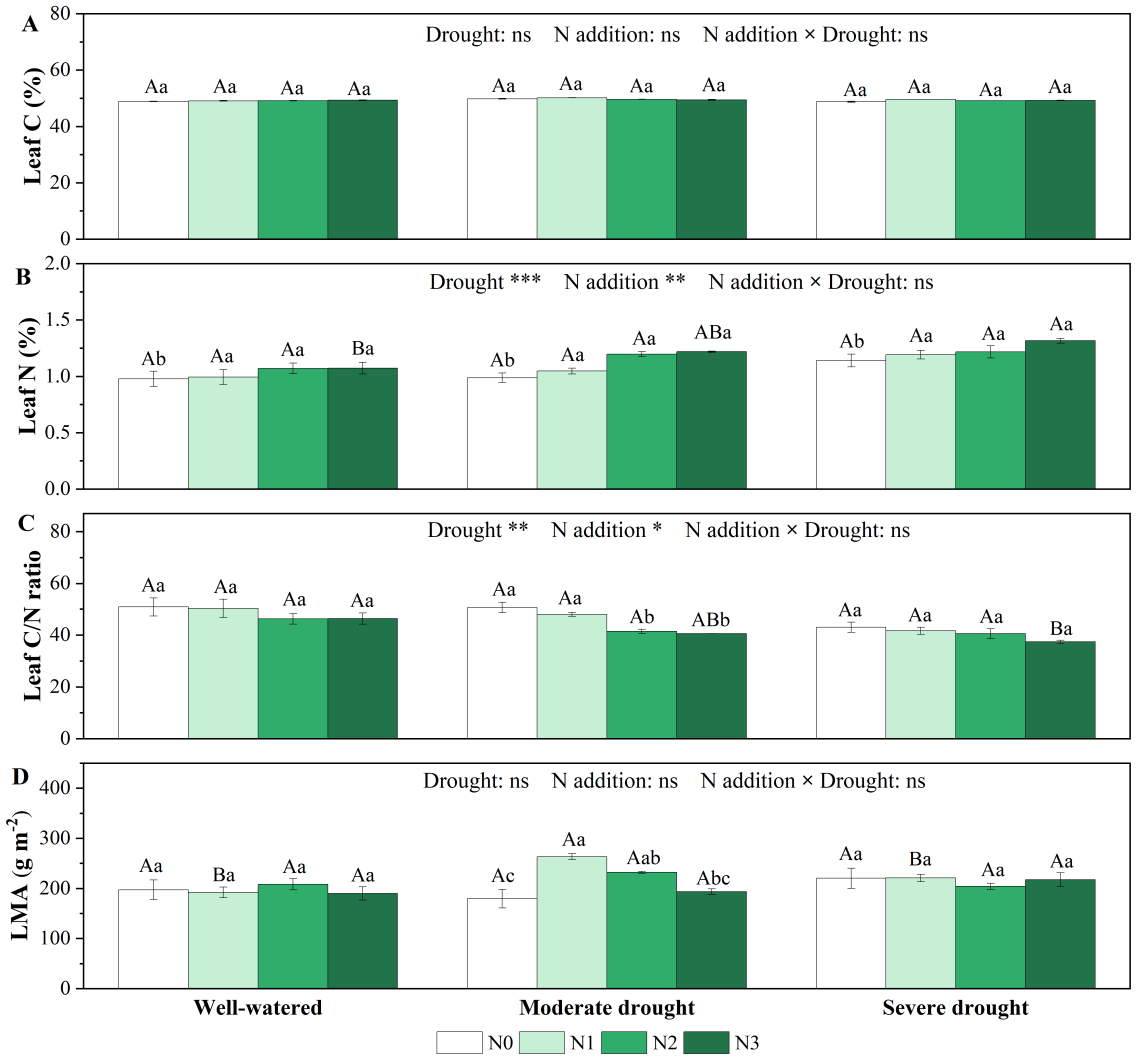
Responses of C content [**(A)**, %], N content [**(B)**, %], C/N ratio in the needles **(C)** and leaf mass per area [**(D)**, LMA, g m^-2^] of *P. koraiensis* to different treatments of soil water stress and N addition. Different uppercase letters indicated significant differences among different drought treatments at the same N addition level, and different lowercase letters indicated significant differences among different N addition levels in the same drought treatment (*P* < 0.05). n = 3. ***p < 0.001, **p < 0.01, *p < 0.05. N0-N3 represent N addition levels at 0, 23, 46, and 69 kg N ha^-1^ yr^-1^ respectively.

Drought, N addition treatments and their interaction had no significant effect on leaf C content (**Figure 3A**; **Supplementary Table S1**). Single drought treatments did not significantly affect leaf N content, but leaf N content was significantly higher in N1-N3 than in N0 under all three soil moisture conditions (**Figure 3B**). Both single drought and N addition treatments had no significant effect on leaf C/N ratio, while under moderate drought, higher N additions (N2 and N3) significantly decreased C/N ratio (**Figure 3C**). In addition, we found no statistically significant effects of single drought, N addition treatments, or their interaction on stem C and N content (**Supplementary Figure S3**, **Supplementary Table S1**).

The effects of single drought and N addition treatments and their interaction on LMA were not significant (**Figure 3D**; **Supplementary Table S1**). However, under moderate drought, low N addition (N1) and medium N addition (N2) significantly increased LMA and reached a maximum (263.52 ± 5.80 g m^-2^) at low N (N1) addition.

### 3.4 Relationships in tree functional traits

**Figure 4:**
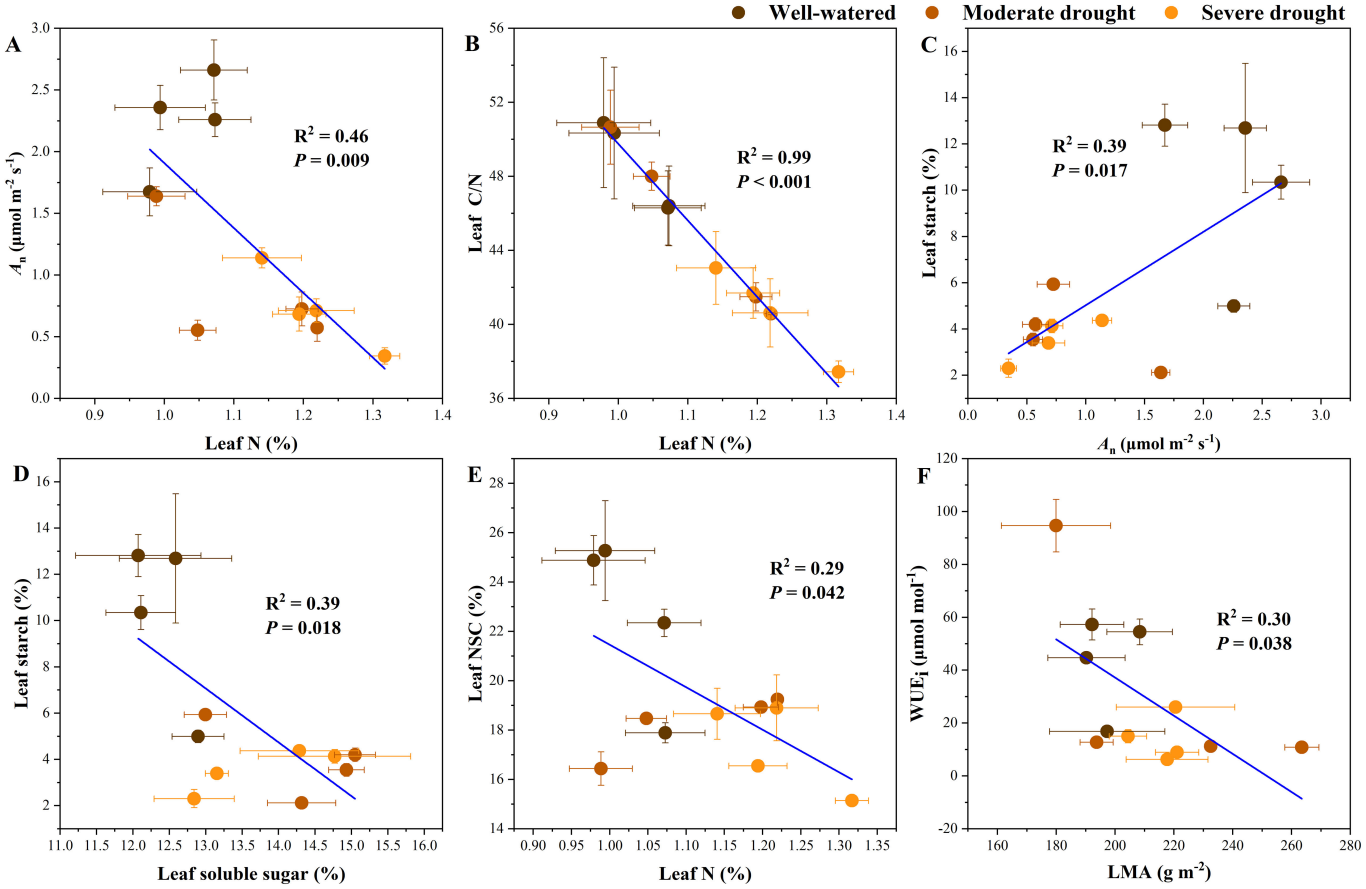
Relationships in key functional traits, including relationships between net photosynthetic rate (*A_n_*) and leaf N content **(A)**; between leaf N content and C/N **(B)**; between leaf starch and *A_n_***(C)**; between leaf starch and leaf soluble sugar **(D)**; and between leaf N content and leaf NSC **(E)**; between the leaf mass per area (LMA) and intrinsic water use efficiency (WUE_i_) **(F)**. The error bar represents SE of all measurements.

Through regression analyses we found significant relationships between key functional traits (**Supplementary Figure S4**). Significant linear relationships were found among *A*_n_, leaf N, leaf C/N, leaf starch, leaf soluble sugar, leaf NSC, WUE_i_ and LMA, specifically reflecting the effects of drought, N addition, and their interactions on carbon assimilation and reserves (**Figure 4**). *A*_n_ (*R* ^2^ = 0.46, *P* = 0.009) and leaf C/N ratio (*R* ^2^ = 0.99, *P* < 0.001) were significantly reduced when leaf N content increased (**Figures 4A, B**). Leaf starch concentrations showed a significant positive correlation with *A*_n_ (*P* = 0.017, *R* ^2^ = 0.39) and a negative correlation with leaf soluble sugar concentrations (*R* ^2^ = 0.39, *P* = 0.018) (**Figures 4C, D**). NSC concentrations decreased with increasing N content (*P* = 0.042, *R* ^2^ = 0.29; **Figures 4E**). This indicated that the increase in leaf N content caused a reduction in carbon assimilation, resulting in changes in carbon reserves. Additionally, LMA is significantly negatively correlated with WUE_i_ (*R* ^2^ = 0.30, *P* = 0.038; **Figures 4F**).

## 4 Discussion

### 4.1 Responses of carbon assimilation to drought and nitrogen addition

In the study, the effects of single drought and N treatments on gas exchange traits (such as *A*_n_ and *g*_s_) of Korean pine were obviously different. When soil water availability decreases, plants can reduce water loss by closing stomata, leading to the decrease in *A*_n_. The reduction in *g*_s_ is the main cause of gas exchange decrease (stomatal limitation). Under severe water stress (severe drought), the epidermal cells surrounding the stomata lose water more rapidly than the guard cells. This imbalance in water loss can cause stomatal dysfunction, leading to the “passive opening” of stomata. This occurs because the guard cells, which are unable to maintain turgor, fail to close properly. As a result, the photosynthetic system, including key enzymes like Rubisco, becomes damaged, leading to a decrease in *A*_n_ due to non-stomatal limitations (Jones, 1985; Novick et al., 2015). Then *A*_n_ reaches a minimum and *g*_s_ appears to increase (**Figures 1A, B**), which is why WUE_i_ reached a maximum under moderate drought in the study (**Figure 1C**). Similar response to single water stress was also observed with other coniferous species, such as *Pinus sylvestris* (Marozas et al., 2019) and *Pinus pinaster* seedlings (de Miguel et al., 2012).

Single N addition treatments, on the other hand, led to a significant increase in *A*_n_ of Korean pine but a decrease in its *g*_s_, resulting in a significant increase in its WUE_i_ (**Figure 1**). A study on Chinese fir (*Cunninghamia lanceolata*) seedlings revealed a nonlinear photosynthetic response to N deposition, which was determined by alterations in photosynthetic capacity rather than stomatal conductance (Li et al., 2022). The promotion of leaf photosynthesis by N addition may benefit from changes in the content of enzymes closely related to photosynthesis (Cornwell et al., 2018).

However, with combined drought and N addition treatments, N addition failed to mitigate the negative effects of drought on carbon assimilation for Korean pine. Under drought stress (moderate and severe drought treatments), all N addition levels significantly reduced *A*_n_ and WUE_i_, although N1 and N2 significantly increased *g*_s_ (**Figures 1A–C**). Similar results were reported for the coniferous species of *Picea asperat* (Tang et al., 2017). Under drought, added N dissolves in available soil water and may cause additional water stress (van den Driessche et al., 2003). N addition can cause a shift in biomass allocation pattern (in favor of above-ground biomass) by increasing in biomass stem: root ratio, which further increases the evapotranspiration demand of plants, leading to higher drought sensitivity (Meyer-Grünefeldt et al., 2015). In contrast to coniferous species, N addition mitigates the negative effects of drought by enhancing carbon exchange in angiosperm species (Zhang et al., 2021a). Broadleaf angiosperm species have higher vein density than coniferous species, which will increase the area of leaf water exchange with the phyllocytes (Sack and Holbrook, 2006). In addition, angiosperms have higher fine root density and volume than conifers and have a greater ability to access water in the soil (Wang C. et al., 2019). Furthermore, the tracheids of coniferous species are always less efficient at water transport than the vessels of broadleaf angiosperm species under sufficient water supply (Sperry et al., 2006).

Moreover, C/N represents the ability of plants to assimilate C when absorbing N, reflecting the efficiency of nutrient use. Under the combined stress, *A*_n_ and C/N decreased with the increase of leaf N content (**Supplementary Figures S4**, **4A, B**), indicating that *P. koraiensis* saplings did not efficiently made use of the nutrients. We also observed a negative correlation between WUE_i_ and LMA (**Supplementary Figures S4**, **4F**). At moderate drought, where WUE_i_ reached maximum values under N0 treatment, N addition (N1-N3) resulted in an increase in LMA (**Figure 1C**). Some studies stated that lower LMA indicates lower leaf construction costs associated with a rapid growth strategy (Wright et al., 2001, 2005). This infers that *P. koraiensis* saplings adopt a conservative strategy by increasing LMA to offset the decrease in water use efficiency (**Figure 3D**), thereby adapting better to the deterioration of water conditions.

### 4.2 Response of carbon reserves to drought and nitrogen addition

Although we found that all treatments of single drought and N addition reduced the leaf NSC, the effecting mechanisms of drought and N addition stress should be different. Generally, drought stress causes a reduction in carbon assimilation, and then stored NSC is mobilized, and starch stored in leaves undergoes hydrolysis to maintain cell expansion and sustain metabolic activity (McDowell et al., 2008; McDowell, 2011), which is also supported by our study (**Figure 2**). During single N addition treatments, N addition increased carbon assimilation by enhancing the net photosynthetic rate, but the newly assimilated carbon is preferentially used for growth and respiration rather than carbon storage (Li et al., 2020). A recent meta-analysis demonstrated that the NSC in terrestrial plants respond differently to different environmental stress (Du et al., 2020). For example, the change of soluble sugars often occurs during N addition and drought, while starch changes with elevated CO_2_ and warming (Du et al., 2020). However, we found similar soluble sugar contents with different treatments in the study. This may be a protective effect of its own (**Figure 2A**), maintaining a certain osmotic regulation ability to prevent leaves from excessively losing water (Liu et al., 2004). However, different from our results on coniferous species, leaf NSC and its component concentrations of two broadleaf species (*Quercus variabilis* and *Liquidambar formosana*) did not change significantly under single N and water treatment or their combination (Jiang et al., 2022).

We found that N addition significantly increased starch concentrations compared with N0 under moderate drought, indicating that sufficient N addition can slow down the hydrolysis of starch into soluble sugars (**Figure 2B**), which may be related to osmoregulation. Previous studies suggested that reduced N-supply under drought stress decreases the content of N-containing osmoprotectants such as proline, which positively affects enzyme and membrane integrity (Ashraf and Foolad, 2007; Gessler et al., 2017). N addition can improve plant water status through osmoregulatory mechanisms, by reducing the increase rate of malondialdehyde content (MDA) and alleviating damage to the plant cell membrane (Liu et al., 2022). For example, it has been demonstrated that under drought stress, N addition significantly reduced the MDA content in three subtropical broadleaf plants (*Schima superba*, *Castanopsis fissa*, and *Michelia macclurei*) (Zhang et al., 2020) and conifer (*Pinus massoniana*) seedlings (Wang D. et al., 2019) to ameliorate the negative effects of drought. However, N addition cannot change the tendency of drought stress to inhibit plant physiological metabolism, but can only slow down the inhibition caused by water shortage. More serious excessive N addition is detrimental to the improvement in the enzymatic defense system of plant cells, leading to leakage of cellular electrolytes and a large increase in the membrane lipid peroxidation product MDA (Su et al., 2021).

Differing from coniferous species, studies on broadleaf species suggested no significant changes in leaf NSC with combined N addition and drought treatments (Zhang et al., 2021a). There may be several reasons to explain this. Firstly, the leaf structure and phloem anatomical features of coniferous species indicated that the outward flow of assimilates in vascular tissues and the rate of phloem transport are lower than in broadleaf species (Dannoura et al., 2011; Liesche and Schulz, 2018). Secondly, for coniferous trees, the imbalance between phloem loading (which may decrease with drought-induced reduction in net CO_2_ assimilation) and unloading, as well as reduced transpiration due to stomatal closure, would reduce the hydrostatic pressure gradient along the phloem and thus reduce the transport rate (Dannoura et al., 2011). In this study, the reduction in *A*_n_ and *g*_s_ exacerbated the reduction in assimilate transport rate, leading to lower NSC reserves ultimately under the combination of drought stress and N addition (**Figure 2B**). The reduced photosynthetic rate required hydrolysis of NSC components to maintain metabolic processes of plants, which was reflected in the relationship between *A*_n_ and starch and the relationship between starch and soluble sugar (**Supplementary Figures S4**, **4C, D**). It indicates that the interconversion between NSC components can regulate the carbon acquisition and expenditure in coniferous species.

### 4.3 Coordination mechanisms of carbon assimilation and reserves during drought and nitrogen addition

Drought had a negative effect on carbon assimilation, while the opposite was found for N addition. Combination treatments did not improve *A*_n_ reduction despite *g*_s_ increase; the WUE_i_ of *P. koraiensis* saplings was as well significantly reduced. Both drought and N addition significantly reduced the total NSC concentrations in leaves, which was caused by the decrease in starch concentrations, although the underlying mechanisms were different. Under the combined treatments, the starch concentrations were significantly increased, yet this only occurred under moderate drought conditions, indicating that different degrees of drought stress and N addition have distinct influences on gas exchange and carbon reserves. Hence, we summarize the physiological mechanisms by which *P. koraiensis* saplings respond to drought stress and N addition. Under drought stress, closure of stomata will lead to reductions in photosynthesis, hydrolysis of starch and conversion to soluble sugars to maintain plant osmotic pressure and metabolism. N addition can increase photosynthetic rate, and newly assimilated carbon will be invested in growth and respiration preferentially over NSC storage. Under drought with N addition, *P. koraiensis* will maintain carbon and water fluxes by increasing stomatal conductance, but will fail to prevent *A*_n_ reduction, leading to a decrease in carbon assimilation. As a result of the decrease in carbon assimilation, stored starch underwent hydrolysis and stored NSC will be mobilized.

### 4.4 Future work

To better understand the physiological adaptation mechanisms underlying interactive stresses of drought and N deposition, further work should take into account: (1) In this study, we used N per unit mass but not N per unit area to analyze the leaf C and N contents, which may to some extent ignore the effects of leaf thickness and density (Rosati et al., 2000). (2) Integrate intrinsic regulatory mechanisms in different plant parts for more detailed comprehensive analysis, including migration between different aboveground and belowground organs, as well as responses of anatomical structures, hydraulic properties and eventually biomass; (3) More finely time scale measurements would be helpful to understand the diel and seasonal responses of conifer trees, encompassing different growth and development periods.

## 5 Conclusions

In this study, we investigated how drought and N addition stresses affect carbon assimilation and carbon reserves of *P. koraiensis* saplings. We found that N addition was not able to mitigate all the negative effects of drought stress on carbon assimilation. The interconversion between leaf NSC components can slow down the reduction of carbon assimilation, which was at the expense of carbon reserves. Our study indicated that *P. koraiensis* failed to benefit from N investment and adapted to reduced water use efficiency by increasing LMA, which suggests a conservative resource use strategy.

## Data Availability

The raw data supporting the conclusions of this article will be made available by the authors, without undue reservation.
