# Supplementary material for: Unravelling the combined effects of drought and nitrogen addition on carbon assimilation and reserves in Korean pine saplings
Source: Front Plant Sci. 2025 Apr 14;16:1574468. doi: 10.3389/fpls.2025.1574468 (PMC12034718; doi:10.3389/fpls.2025.1574468)
Supplement: Supplementary file 1 [file DataSheet1.pdf]

Supplementary Material

1 Supplementary Figures and Tables

1.1 Supplementary Figures

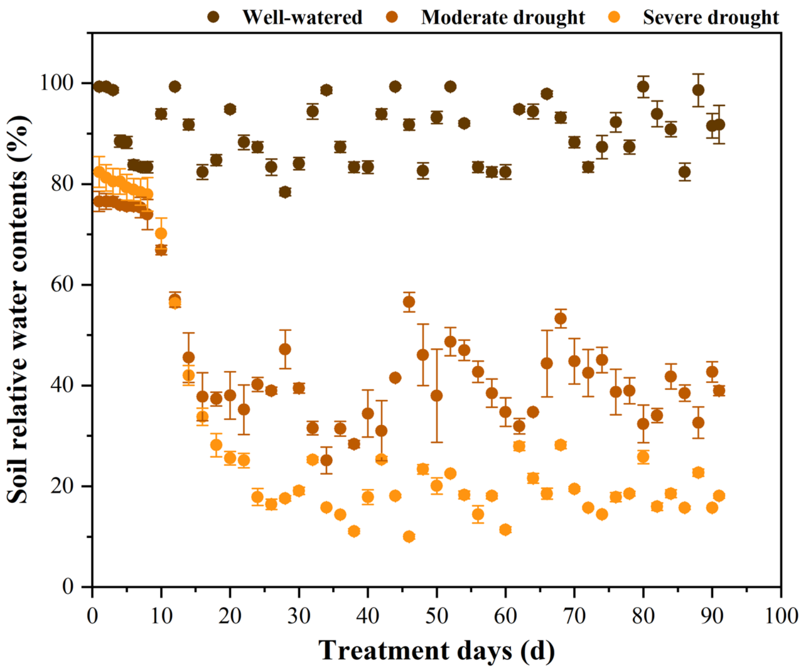

Supplementary Figure 1. The change of soil water condition during experimental period.

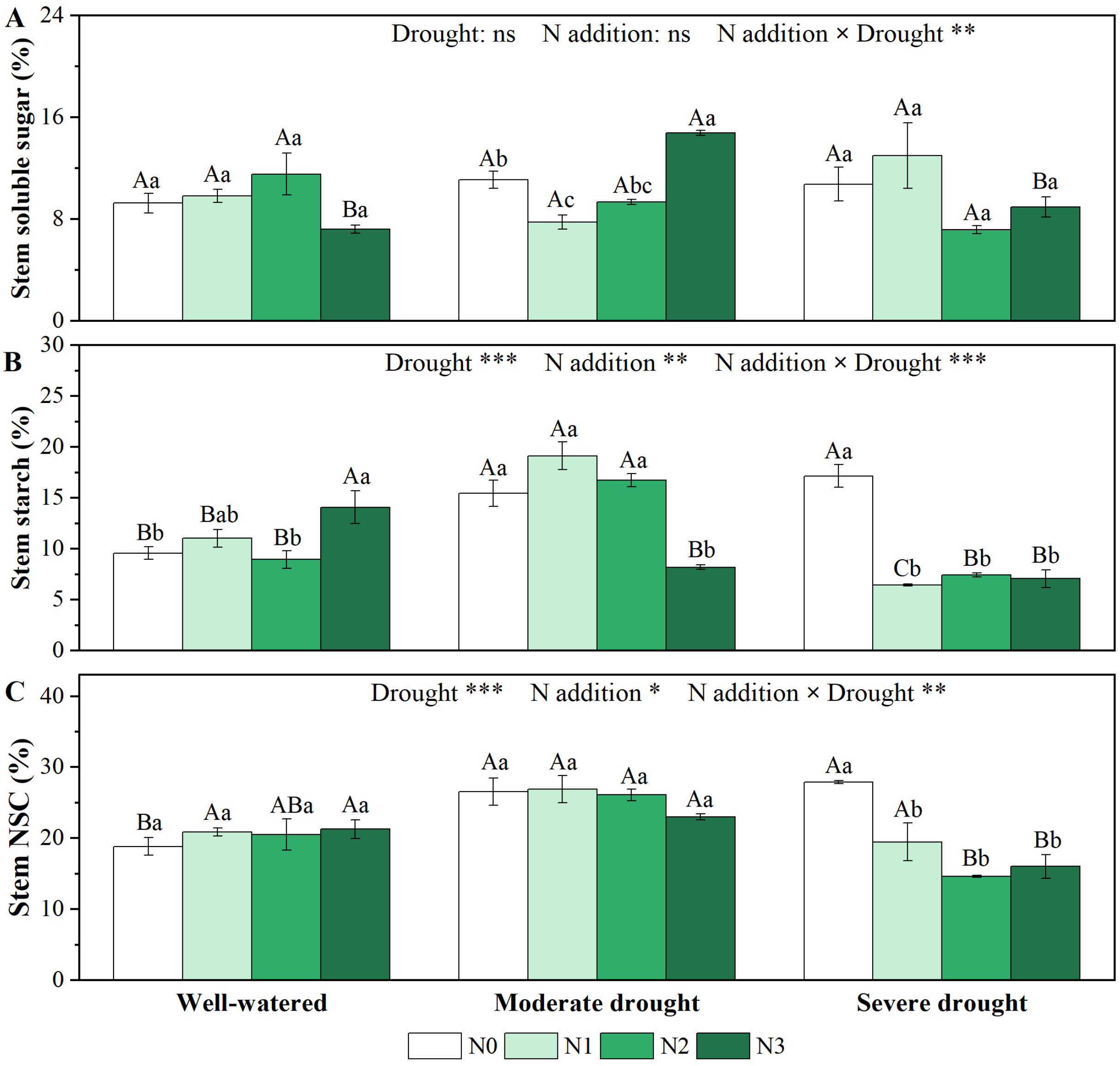

Supplementary Figure 2. Responses of soluble sugar (%), starch (%), and non-structural carbohydrates (NSC, %) in the stems of *P. koraiensis* to different treatments of soil water stress and N addition. Different uppercase letters indicated significant differences among different drought treatments at the same N addition level, and different lowercase letters indicated significant differences among different N addition levels in the same drought treatment ($P < 0.05$). n = 3. ***$p <$

0.001, **$p < 0.01$, *$p < 0.05$. N0-N3 represent N addition levels at 0, 23, 46, and 69 kg N ha^{-1} yr^{-1} respectively.

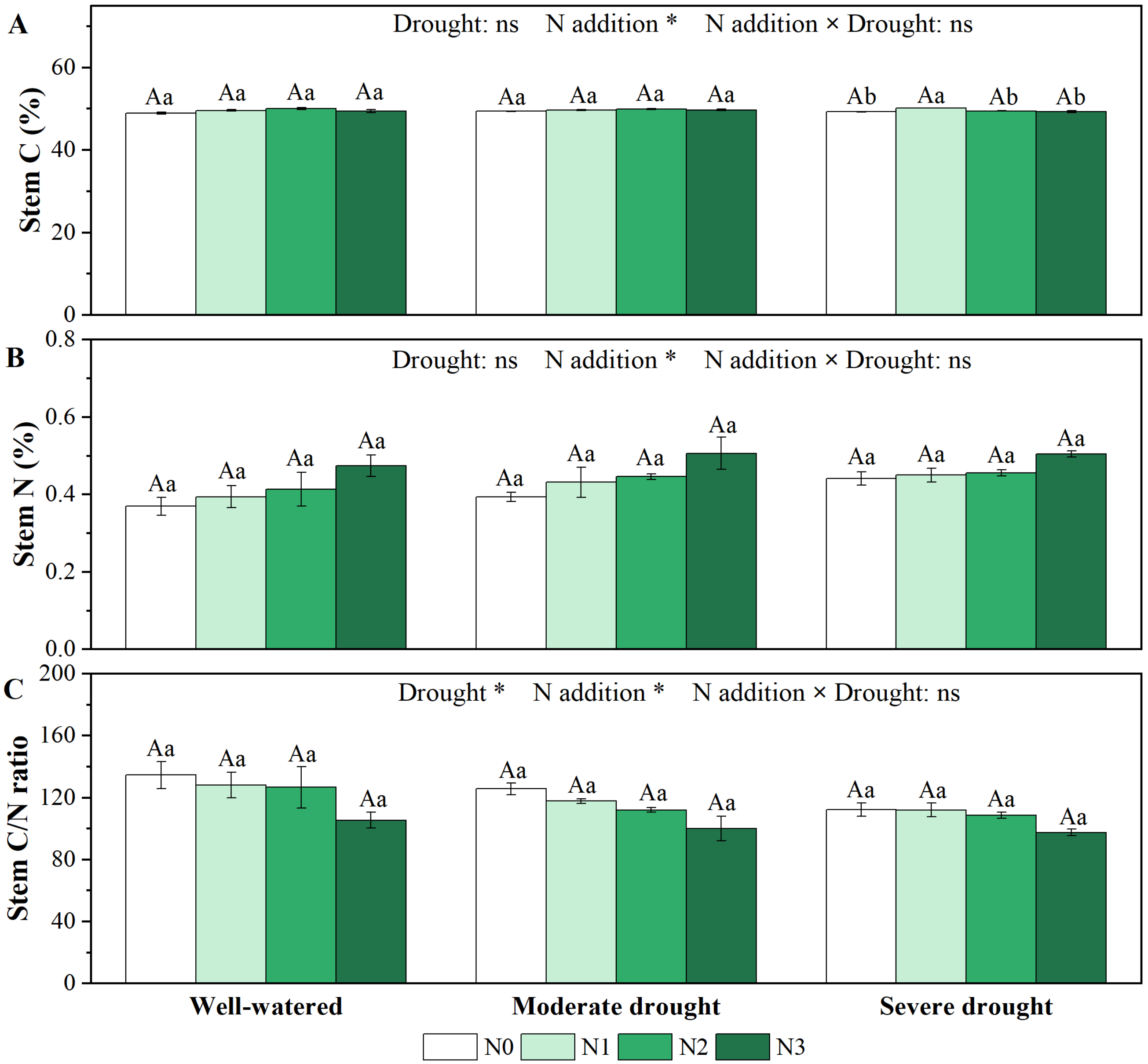

Supplementary Figure 3. Responses of C content (%), N content (%), C/N ratio in the stems of *P. koraiensis* to different treatments of soil water stress and N addition. Different uppercase letters indicated significant differences among different drought treatments at the same N addition level, and different lowercase letters indicated significant differences among different N addition levels in the same drought treatment ($P < 0.05$). n = 3. ***$p < 0.001$, **$p < 0.01$, *$p < 0.05$. N0-N3 represent N addition levels at 0, 23, 46, and 69 kg N ha^{-1} yr^{-1} respectively.

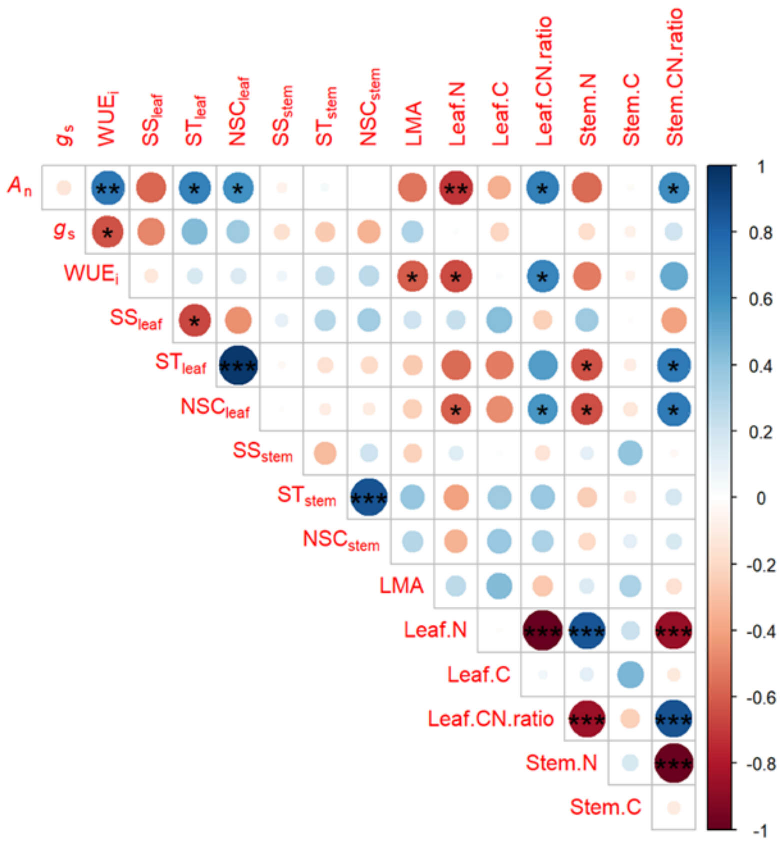

Supplementary Figure 4. Correlation in key tree functional traits. Traits are net photosynthetic rate (A_n), stomatal conductance (g_s), intrinsic water use efficiency (WUE_i), leaf soluble sugar concentrations (SS_{leaf}), leaf starch concentrations (ST_{leaf}), leaf non-structural carbohydrates concentrations (NSC_{leaf}), stem soluble sugar concentrations (SS_{stem}), stem starch concentrations (ST_{stem}), stem non-structural carbohydrates concentrations (NSC_{stem}), leaf N content (N_{leaf}), leaf C content (C_{leaf}), leaf C/N ratio (C/N_{leaf}), stem N content (N_{stem}), stem C content (C_{stem}), stem C/N ratio (C/N_{stem}) and leaf mass per area (LMA). The color of each circle represents the correlation coefficient, and the size of the circle represent the significance level (***$P < 0.001$, **$P < 0.01$, *$P < 0.05$).

1.2 Supplementary Tables

Table S1. Results of two-way ANOVA showed the effects of N addition, drought stress and their interaction. A_n: net photosynthetic rate; g_s: stomatal conductance; WUE_i: intrinsic water use efficiency; SS_{leaf}: leaf soluble sugar concentrations; ST_{leaf}: leaf starch concentrations; NSC_{leaf}: leaf non-structural carbohydrates concentrations (SS_{leaf} + ST_{leaf}); SS_{stem}: stem soluble sugar concentrations; ST_{stem}: stem starch concentrations; NSC_{stem}: stem non-structural carbohydrates concentrations (SS_{stem} + ST_{stem}); N_{leaf}: leaf N content; C_{leaf}: leaf C content; C/N_{leaf}: leaf C/N ratio; N_{stem}: stem N content; C_{stem}: stem C content; C/N_{stem}: stem C/N ratio; LMA: leaf mass per area. n = 3. Significance at $P < 0.05$ is presented in bold.

Dependent variable	N addition			Drought			N addition × Drought		
	df	*F*	*P*	df	*F*	*P*	df	*F*	*P*
A_n	3	2.357	0.074	2	87.048	**<0.001**	6	6.607	**<0.001**
g_s	3	4.304	**0.006**	2	31.078	**<0.001**	6	76.927	**<0.001**
WUE_i	3	11.375	**<0.001**	2	27.987	**<0.001**	6	35.813	**<0.001**
SS_{leaf}	3	0.113	0.952	2	7.291	**0.003**	6	1.726	0.158
ST_{leaf}	3	4.755	**0.010**	2	45.786	**<0.001**	6	4.493	**0.003**
NSC_{leaf}	3	4.601	**0.011**	2	28.229	**<0.001**	6	4.980	**0.002**
SS_{stem}	3	0.416	0.743	2	1.088	0.351	6	5.445	**0.001**
ST_{stem}	3	7.226	**0.001**	2	20.916	**<0.001**	6	15.916	**<0.001**
NSC_{stem}	3	3.723	**0.023**	2	12.758	**<0.001**	6	4.280	**0.004**
N_{leaf}	3	4.996	**0.007**	2	10.615	**<0.001**	6	0.458	0.833
C_{leaf}	3	2.096	0.133	2	0.224	0.801	6	0.685	0.664
C/N_{leaf}	3	4.080	**0.016**	2	8.568	**0.001**	6	0.384	0.883
N_{stem}	3	4.304	**0.013**	2	2.447	0.102	6	0.111	0.994
C_{stem}	3	4.001	**0.017**	2	0.419	0.662	6	1.285	0.296
C/N_{stem}	3	3.613	**0.025**	2	3.376	**0.049**	6	0.157	0.986
LMA	3	1.750	0.177	2	2.263	0.120	6	1.929	0.106
